# Downregulation of ATP1A1 Expression by *Panax notoginseng* (Burk.) F.H. Chen Saponins: A Potential Mechanism of Antitumor Effects in HepG2 Cells and *In Vivo*

**DOI:** 10.3389/fphar.2021.720368

**Published:** 2021-10-07

**Authors:** Xiao-Yi Feng, Wei Zhao, Zheng Yao, Ning-Yi Wei, An-Hua Shi, Wen-Hui Chen

**Affiliations:** Faculty of Basic Medicine, Yunnan University of Chinese Medicine, Kunming, China

**Keywords:** Na+/K+-ATPase α1 subunit, *Panax notoginseng* saponins, digitonin, antitumor, AKT/ERK signaling pathways

## Abstract

The Na^+^/K^+^-ATPase α1 subunit (ATP1A1) is a potential target for hepatic carcinoma (HCC) treatment, which plays a key role in Na^+^/K^+^ exchange, metabolism, signal transduction, etc. *In vivo*, we found that *Panax notoginseng* saponins (PNS) could inhibit tumor growth and significantly downregulate the expression and phosphorylation of ATP1A1/AKT/ERK in tumor-bearing mice. Our study aims to explore the potential effects of PNS on the regulation of ATP1A1 and the possible mechanisms of antitumor activity. The effects of PNS on HepG2 cell viability, migration, and apoptosis were examined *in vitro*. Fluorescence, Western blot, and RT-PCR analyses were used to examine the protein and gene expression. Further analysis was assessed with a Na^+^/K^+^-ATPase inhibitor (digitonin) and sorafenib *in vitro*. We found that the ATP1A1 expression was markedly higher in HepG2 cells than in L02 cells and PNS exhibited a dose-dependent effect on the expression of ATP1A and the regulation of AKT/ERK signaling pathways. Digitonin did not affect the expression of ATP1A1 but attenuated the effects of PNS on the regulation of ATP1A1/AKT/ERK signaling pathways and enhanced the antitumor effect of PNS by promoting nuclear fragmentation. Taken together, PNS inhibited the proliferation of HepG2 cells *via* downregulation of ATP1A1 and signal transduction. Our findings will aid a data basis for the clinical use of PNS.

## Introduction

The Na^+^/K^+^-ATPase sodium pump (NKA) is widely distributed in mammalian cell membranes and participates in Na^+^/K^+^ exchange, maintaining ion balance and cellular osmotic pressure (Lan et al., 2018). The NKA consists of α1, α2, α3, α4, β1, β2, β3, and γ subtypes (Chen et al., 2006). The α subunit includes the binding sites of ATP, cardiac glycosides (CTS), Na^+^, K^+^, and ouabain (Lingrel et al., 2007). A recent study reported that any mutations in the NKA gene may cause serious physiological disturbances compared to the inactivation of ion pump function. Such physiological disturbances may be caused by leakage of ion channels, protein instability, or misfolding (Sweadner et al., 2019). The α1 subunit (ATP1A1) is overexpressed in liver cancer (Zhuang et al., 2015), glioma (Xu et al., 2010), etc. Downregulating the expression of ATP1A1 can significantly reduce the proliferation and migration of hepatic carcinoma (HCC) cells and promote cell apoptosis, reducing their tumorigenicity *in vivo*. The change in the α subunit will disrupt the homeostasis of the Src family of protein kinases (Src). Activated Src can bind to the epidermal growth factor receptor (EGFR) to induce subsequent activation of phosphoinositide 3-kinase (PI3K), Ras/Raf/ERK, and PLC/PKC. Meanwhile, Src can induce the mitochondria to produce a large amount of ROS and upregulate intracellular Ca^2+^ concentration to activate the second messenger (Yu et al., 2019).

*Panax notoginseng* (Burk.) F.H. Chen is widely used in traditional Chinese medicine (TCM). *Panax notoginseng* saponins (PNS) are the major active ingredient of *P. notoginseng* and also the main ingredient of Xuesaitong, Xueshuantong, and other Chinese patent medicines to treat cardio–cerebrovascular diseases. Nowadays, the pharmacological effects of PNS are not limited to the treatment of cardiovascular diseases but also include anti-inflammatory, (Hu et al., 2018; Zhao et al., 2018; Lu et al., 2020), antitumor (Wang et al., 2014; Yang et al., 2016; Li Q. et al., 2020), antithrombotic, and anti-arterial injury properties (Hui et al., 2020). The saponins in *P. notoginseng* are mainly dammarane-type and ocotillol-type. PNS mainly contain five active ingredients: notoginsenoside R_1_, ginsenoside Rg_1_, ginsenoside Re, ginsenoside Rb_1_, and ginsenoside Rd. The five main compounds are dammarane-type saponins. The latest systematic research on the composition of PNS showed that 28 compounds were isolated from PNS, including several rare saponins, and they exhibited antitumor activities (Li Q. et al., 2020). The antitumor mechanisms mainly included promoting cell apoptosis, inhibiting the mTOR/PI3K/AKT signaling pathway (Wei Li et al., 2019), reducing the ERK signaling pathway (Meng et al., 2019), downregulating miR-21 (Li Y. et al., 2020), promoting DNA damage (Cai et al., 2021), etc.

However, these previous reports did not investigate the mechanism of action that led to the downregulation of signaling pathways. In addition, *in vitro* study of PNS on the regulation of cell membrane proteins was very limited. In the present study, we hypothesized that PNS can inhibit AKT/ERK signaling pathways *via* downregulating the ATP1A1 expression. Therefore, we designed the experiment to illustrate the effect of PNS on ATP1A1 in HepG2 cells and explore the possible mechanisms.

## Materials and Methods

### Regents and Chemicals

PNS were purchased from Xi’an TianBao Guangyuan Biotech. Ltd. (Shanxi Province, China, purity > 95%), and they include notoginsenoside R_1_>5.0%, ginsenoside Rg_1_>25.0%, ginsenoside Re >2.5%, ginsenoside Rb_1_>30.0%, and ginsenoside Rd >5.0% (Pharmacopoeia of the People’s Republic of China (2020)). A Dulbecco’s Modified Eagle Medium (DMEM) with high glucose was brought from Gibco (United States, 1980922). The ATP1A1 inhibitor (digitonin, ST1272) was brought from Biotech (Beijing, China). Sorafenib (SC0236) was purchased from Beyotime, China. The Servicebio^®^ RT First Strand cDNA Synthesis Kit (G3330) and 2×SYBR Green qPCR Master Mix (High ROX) (G3322) were obtained from Servicebio. TRIzol and RAPA were used in this study. ATP1A1, ERK, and p-ERK antibodies were purchased from Cell Signaling Technology (United States; batch numbers are 23565S-1, 4695S-14, 9101S-28); mTOR, p-mTOR, AKT, and p-AKT were purchased from Abcam (Abcam, United Kingdom; batch numbers are GR3181969-14, GR112975-29, GR43522-33, and GR297104-1). β-Actin antibody (Wuhan Servicebio Company; lot number: 180926), FITC rabbit antibody (Beijing Zhongshan Jinqiao Biotechnology Co., Ltd.; lot number: 133027), and DAPI (Beijing Beyotime Biotechnology Co., Ltd.; lot number: C1005) were also used.

### Cell Culture and Animals

HepG2 cells, HL-7702 (L-02) normal human liver (L02) cells, and H22 cells were incubated in a DMEM (Gibco) supplemented with 10% fetal bovine serum (Gibco), 10,000 U/mL penicillin, and 1% streptomycin. HepG2 cells (5 × 10^5^ cells/well) were incubated in 12-well plates for 24 h. Then, cells were treated with different doses of PNS (600 mg/L and 300 mg/L, resp.) and incubated for 48 h. C57bl/6 mice (male, body weight 16 ± 2 g) were purchased from Hunan STA Laboratory Animal Co., Ltd. (Changsha, China, SCXK (Xiang) 2016-0002). The procedures of animal studies were designed according to the national and international guidelines and approved by the Committee of the Yunnan University of Chinese Medicine (SYXK (Dian) K2017-0005).

### Antitumor Evaluation *In Vivo*

A total of 2 × 10^6^ H22 cells/200 μl were injected into mice by subcutaneous injections to establish a tumor growth model. Mice were weighed, and the tumor volumes were measured once every 2 days according to the formula (A × B^2^)/2 (A: the tumor length and B: the tumor width). After the tumor volumes reached 50–100 mm^3^, they were randomly divided into four groups and treated with normal saline, sorafeinb (60 mg/kg), and different dosages of PNS by intragastric administration once per day, for 14 days, with 6 mice/each group. The doses of PH (high dosage of PNS) and PL (low dosage of PNS) groups were 100 mg/kg/day and 50 mg/kg/day, respectively. The control groups were given the same volume of normal saline.

### Cell Viability Assay

HepG2 cells (1 × 10^4^ cells/well) were incubated in 96-well plates in a complete DMEM. Cells were treated with different concentrations of PNS (0, 18.75, 37.5 75, 150, 300, and 600 mg/L) for 48 h. Cell viability was determined by the MTT assay. 20 μl MTT was added to each well and incubated for 3 h. The formazan was dissolved in 150 μl of dimethyl sulfoxide. The optical density was measured at 490 nm, and cell viability was normalized as the percentage of control. Cell viability (%)=(OD_control−_OD_sample_)/(OD_control−_OD_blank_) × 100%. IC_50_ (half-maximal inhibitory concentration) was calculated by SPSS software.

### Cell Migration Assay

HepG2 cells (8 × 10^4^ cells/well) were incubated in 24-well plates in a complete DMEM. Using a sterile 200 µl pipette tip, a scratch of 1 mm width was made in triplicate. Floating cells were removed by washing with phosphate buffer solution (PBS), and then, fresh media or PNS were added. Cells were treated with 300 mg/L or 600 mg/L of PNS for 48 h. Images were recorded using an inverted microscope (Olympus, CK40, Japan). The images from 0 to 48 h were compared, and the migration distance was calculated by ImageJ software.

### Annexin V-FITC/PI Staining

HepG2 cells (5 × 10^5^ cells/well) were incubated in 6-well plates. Cells were treated with 300 mg/L or 600 mg/L of PNS for 24 h. Then, the cells were stained with Annexin V/PI for 30 min at 4°C in the dark, and the apoptosis analysis was carried out by flow cytometry (Accuri C6, BD, CA, United States). The apoptotic rate was calculated by FlowJo software version 10 (Ashland, OR, United States).

### RNA Isolation and Real-Time Quantitative PCR Analysis

Total mRNA was obtained from HepG2 cells using the TRIzol reagent, and cDNA was obtained using a cDNA synthesis kit. The primer sequences are listed in Table 1. According to the manufacturer’s protocol, Q-PCR was performed in a real-time PCR system. The 2^−ΔΔ^CT method was used for quantitative analysis, and results were normalized with β-actin.

**TABLE 1:** Primer sequences of target genes.

Target gene	Primer sequences (5’→3′)
ATP1A1 (forward)	GGC​AGT​GTT​TCA​GGC​TAA​CCA​G
ATP1A1 (reverse)	TCT​CCT​TCA​CGG​AAC​CAC​AGC​A
AKT (forward)	CCG​CCT​GAT​CAA​GTT​CTC​CT
AKT (reverse)	TTC​AGA​TGA​TCC​ATG​CGG​GG
ERK (forward)	GCT​CTG​CTT​ATG​ATA​ATC​TC
ERK (reverse)	GATGCCAATGATGTTCTC
β-Actin (forward)	TGA​GCT​GCG​TTT​TAC​ACC​CT
β-Actin (reverse)	GCC​TTC​ACC​GTT​CCA​GTT​TT

### Inhibitor Treatment

To clarify the effects of PNS on ATP1A1 and signaling pathways in HepG2, the Na^+^/K^+^-ATPase inhibitor (digitonin) was used in the experiment. The treatment was divided into five groups, including 10 nmoL/L digitonin group, 10 nmoL/L digitonin combined with 600 mg/L PNS group, 10 nmoL/L digitonin combined with 300 mg/L PNS group, 600 mg/L PNS group, and 300 mg/L PNS group. After 48 h of drug treatment, the proteins of HepG2 cells were detected by Western blot.

### Colony Formation Assay

HepG2 cells (600 cells/well) were incubated in 6-well plates and were treated with 600 and 300 mg/L PNS. One week later, visible colonies were formed. Then, the colonies were fixed with 4% polymethanol and stained with crystal violet (0.005%). The number of colonies was calculated using a microscope.

### Hoechst Staining Assay

HepG2 cells were incubated in 6-well plates and treated with PNS (600 mg/L and 300 mg/L) for 48 h. The cells were then incubated with 500 μl/well of Hoechst stain (Beyotime, Nanjing, China) for 15 min at room temperature. The cells were observed under a fluorescence microscope (Nikon, Japan).

### Fluorescence

Cells (4 × 10^5^/well) were incubated in a 12-well plate containing cell slides for 24 h and continued to be incubated for 48 h after administration. The cells were washed 3 times with PBS, at room temperature, then 1 ml of 4% paraformaldehyde was added to each well for 30 min, and finally the cells were washed thrice with PBS. After blocking with an antibody-blocking solution (FDB) for 30 min, the cells were incubated with the primary antibody (1:100 dilution) overnight at 4°C and washed 3 times with PBS, and the secondary antibody (FITC-goat anti-rabbit IgG antibody, 1:200-fold dilution) of 200 μl/well was added. The cells were washed 5 times with PBS, incubated with DPAI (100 μl/well) for 10 min at room tenmperature in the dark, and then washed 3 times with PBS. The cells were detected with a laser confocal microscope.

### Western Blot Analysis

Tumor tissues and HepG2 cells were lysed in an RIPA buffer solution containing PMSF and protein inhibitors (Roche, Germany). A nucleic acid and protein microanalyzer (Molecular Devices, United States) was used to determine the protein concentration. The proteins were separated using 10% SDS-PAGE gel and transferred onto PVDF membranes. The membranes were blocked with 5% nonfat milk for 2 h and incubated with primary antibodies at 4°C overnight. The membranes were washed with PBST, and the protein bands were detected by the ECL and were exposed to the TANON gel imager (Shanghai, China). The results were analyzed by ImageJ software. Because the molecular weights of the target proteins were the same, the primary and secondary antibodies were removed using a stripping buffer (P0025N, Beyotime Biotechnology, China) and then the membranes were washed with PBST and finally blocked and incubated with the antibodies for subsequent experiments.

### Molecular Docking

The structure of ATP1A1 (PDB code 3KDP) was utilized as a receptor. The three-dimensional chemical structure of ginsenoside Rg_1_ and ginsenoside Rb_1_ was obtained from ChemDraw 3D software, and they were prepared for ligand binding. All of them were preserved in the PDB format for the following procedures. By optimizing Autodock Vina software, ginsenoside Rg_1_ and ginsenoside Rb_1_ interacted with ATP1A1 at a molecular simulative level. The docking center was set as center_x = −21.896, center_y = −12.893, and center_z = 82.863, respectively, and the number of points was set as 60 individually.

### Statistical Analysis

Data were presented as the mean ± standard deviation (SD). Independent two-sample t-tests were used to compare differences between two groups, and one-way ANOVA with the least significant difference (LSD) test for post hoc comparisons was used to compare differences between groups. P < 0.05 was indicated as statistical significance. Statistical analysis was performed using SPSS 21.0 software (SPSS Inc., Chicago, IL, United States). Figures were constructed using GraphPad Prism 8 software.

## Results

### The Quality Control Results of PNS

**FIGURE 1:**
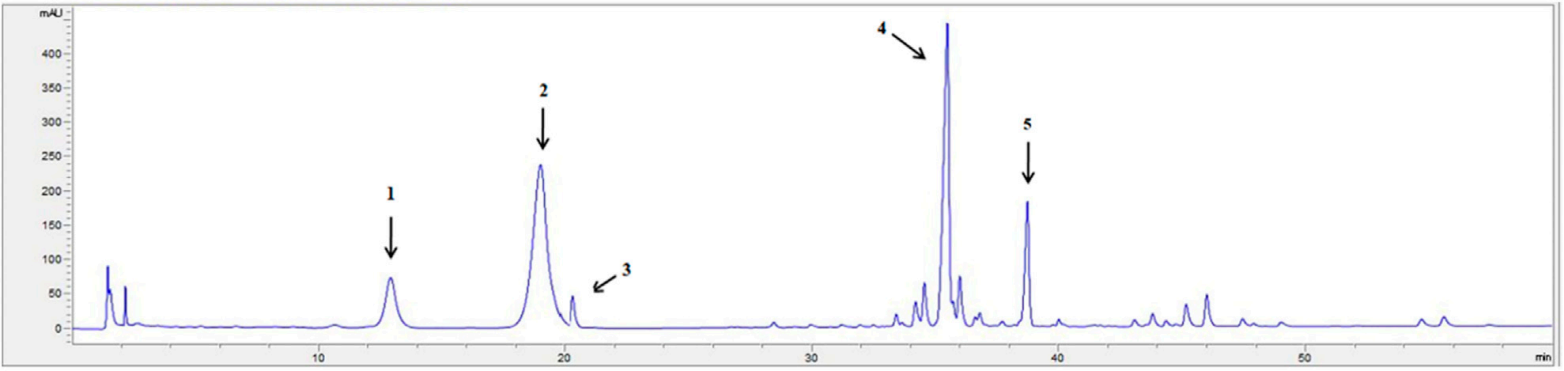
The HPLC fingerprint profile of PNS. 1: notoginsenoside R_1_; 2: ginsenoside Rg_1_; 3: ginsenoside Re; 4: ginsenoside Rb_1_; 5: ginsenoside Rd. The chromatography was performed on an Agilent SB-C18 column (250 × 4.6 mm, 5 μm), at 30°C, with a UV detection rate of λ = 203 nm and flow rate = 1.5 ml/min. Gradient elution: acetonitrile (A) and Water (B): 0 min 20% A, 20 min 20% A, 45 min 46% A, 55 min 55% A, 60 min 55% A. The HPLC chromatogram of PNS was authenticated according to the Pharmacopoeia of the People’s Republic of China identification key (2015, Volume 1).

The HPLC chromatogram of PNS is displayed in Figure 1. The contents of each ingredient in the PNS extract included notoginsenoside R_1_ (10.16 ± 0.31%), ginsenoside Rg_1_ (36.47 ± 1.78%), ginsenoside Re (3.51 ± 0.37%), ginsenoside Rb_1_ (30.79 ± 2.00%), and ginsenoside Rd (9.07 ± 0.25%) and met the Chinese Pharmacopoeia (2015 edition) criterion (Supplementary Figure S1).

### PNS Inhibited Tumor Growth and Suppressed ATP1A1/AKT/ERK Signaling Pathways at the Tumor Sites

**FIGURE 2:**
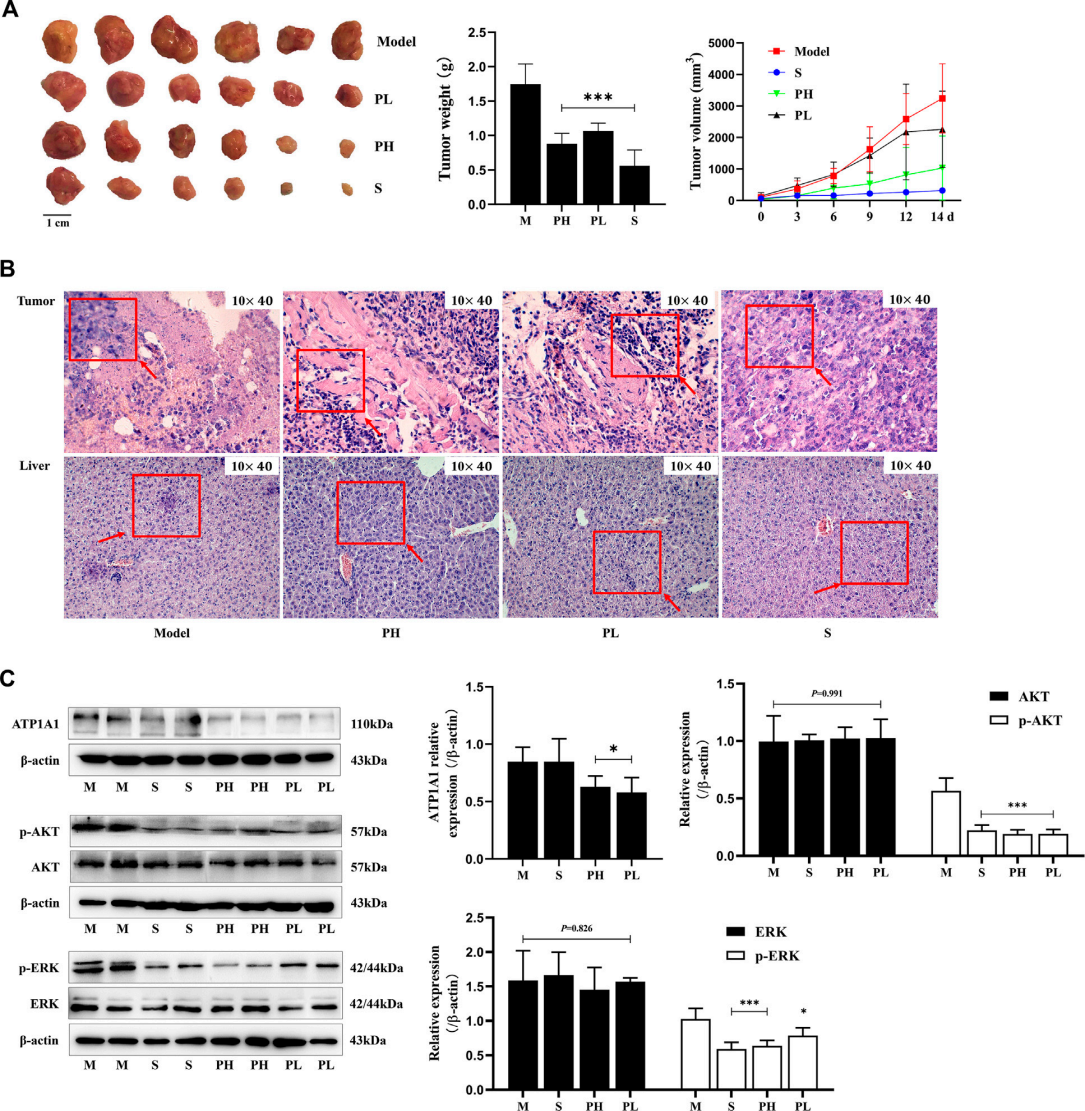
PNS inhibited tumor growth and downregulated the ATP1A1/AKT/ERK signaling pathway. Twenty-four tumor-bearing mice were treated with saline, sorafenib (60 mg/kg), or PNS (50 and 100 mg/kg) by intragastric administration every day for 14 days. **(A)** The morphologies of tumors were photographed, and tumor weights and volumes were measured. **(B)** The HE-stained images of tumors were photographed. **(C)** The Western blot images of ATP1A1, AKT, p-AKT, ERK, and p-ERK and their quantitative analysis are shown. M: saline group; S: sorafenib group; PH: 100 mg/kg PNS group; PL: 50 mg/kg PNS group. Data are expressed as mean ± SD. *p < 0.05, **p < 0.01, and ***p < 0.001 vs. the model group.

PNS inhibited tumor growth in H22 tumor-bearing mice (Figure 2A). 0–7 days after administration, the mice had a better diet and mental state. After 7 days, with the rapid increase in the tumor volume (Figure 2A), the mice’s diet gradually decreased, they were too lazy to exercise, and the amount of feces and urine decreased. The pathological morphology results showed that there was no clear boundary between the tumor and the muscle tissue in the model group and they were completely fused together. The nest-like structure of the tumor tissue in the PH group had obvious boundaries, and the area surrounding the muscle was less. The tumor cells were deeply stained, and the arrangement of cells was crowded and disorderly in the PL group (Figure 2B). As displayed in Figure 2C, ATP1A1 expression and phosphorylation of ERK and AKT were significantly reduced in the PNS and sorafenib groups (*P* < 0.05), but not the expression of ERK and AKT.

### PNS Inhibited the Viability, Migration, and Proliferation of HepG2 Cells

**FIGURE 3:**
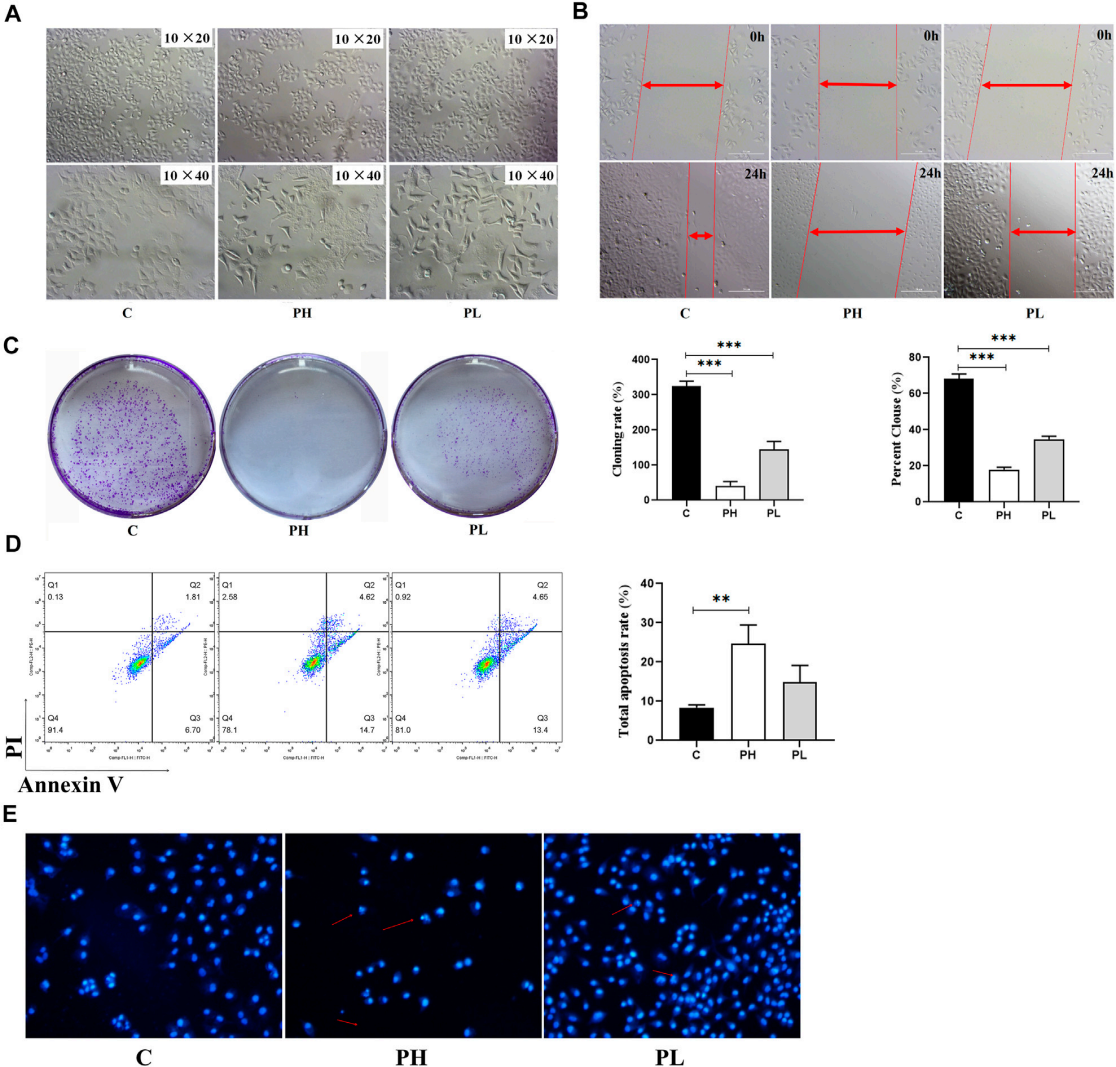
PNS inhibited the proliferation and migration of HepG2 cells. C: control group; PH: high-dose PNS group (600 mg/L); PL: PNS low-dose PNS group (300 mg/L). **(A)** The morphology images of HepG2 cells treated with saline and PNS for 48 h. **(B)** The effect of PNS on the migration of HepG2 cells. **(C)** Giemsa-stained colonies were observed under an inverted microscope. **(D)** FACS analysis of apoptosis in HepG2 cells. The cells were double-stained with Annexin V and PI. Early apoptotic cells were in the lower right quadrant, and late apoptotic cells were in the upper right quadrant. The histogram in the right panel showed average percentages of apoptotic cells which contained the early apoptotic cells and late apoptotic cells. **(E)** Fluorescence images were detected by Hoechst staining. Data are presented as the means ± SD of triplicate experiments. *p < 0.05, **p < 0.01, and ***p < 0.001, vs. the control group.

The MTT assay was used to better evaluate the antitumor viability of PNS (Table 2). The IC_50_ was 124.83 ± 11.24 mg/L. Based on the result, we used two and four times the IC_50_ for experiments as follows (300 mg/L and 600 mg/L). As shown in Figure 3A, the cell viability of HepG2 cells decreased in a dose-dependent manner after PNS treatment. The results of cell migration and colony experiments showed that 300 mg/L of PNS could significantly inhibit the migration of HepG2 cells and the formation of cell clones (Figures 3B,C) and the effect of 600 mg/L was more significant. Flow cytometry and Hoechst staining were used to detect changes in the rate of apoptosis and nuclear condensation and lysis in HepG2 cells (Figures 3D,E). The results showed that compared with the control group, the apoptosis rate of nuclear fragmentation of HepG2 cells increased after PNS treatment for 48 h. PNS inhibited the survival rate and migration rate and increased the apoptosis rate of HepG2 cells.

**TABLE 2:** Effects of PNS on cell viability of HepG2 cells (‾x ± *s*, n = 3).

Group	Concentration (mg•L^−1^)	Cell viability (%)
Control PNS	—	100 ± 0.32
	18.75	79.71 ± 5.23
	37.5	85.43 ± 4.27
	75	64.48 ± 3.21
	150	54.07 ± 2.12
	300	29.25 ± 4.08
	600	8.03 ± 0.94
IC_50_ (mg•L^−1^)		124.83 ± 11.24

### PNS Regulated ATP1A1–Activated AKT, ERK, and PKC Signaling

**FIGURE 4:**
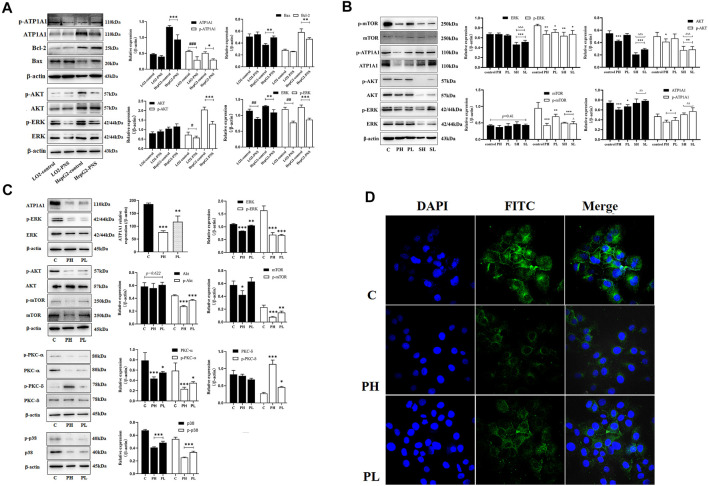
PNS reduced the expression of ATP1A1 and downregulated AKT/ERK signaling pathways. SH: 20 μmoL•L^−1^; SL: 10 μmoL•L^−1^. **(A)** The effects of PNS on regulating the expression of ATP1A1 and AKT/ERK signaling pathways in L02 cells and HepG2 cells. **(B)** The effects of PNS and sorafenib on regulating the expression and phosphorylation of ATP1A1/AKT/ERK signaling pathways. **(C)** The image of proteins after PNS treatment in HepG2 cells. **(D)** The fluorescence image of laser confocal detection of ATP1A1 expression in the HepG2 cell membrane (10 × 60). Scale bar = 50 μm. Data are expressed as mean ± standard deviation. *p < 0.05, **p < 0.01, and ***p < 0.001 vs. the control group; ^#^ *p* < 0.05, ^##^ *p* < 0.01, and ^###^ *p* < 0.001 vs. the L02 control group. ^∆^ *p* < 0.05, ^∆∆^ *p* < 0.01, and ^∆∆∆^ *p* < 0.001 vs. the PNS group.

ATP1A1 expression levels were higher in HepG2 cells than in L02 cells (Figure 4A). PNS could inhibit the expression and phosphorylation of ATP1A1 in both cell lines. We detected the effect of PNS on ATP1A1–relative signaling pathways. Compared with L02 cells, the AKT and ERK signaling pathways were activated in HepG2 cells, especially the expression and phosphorylation of AKT. PNS inhibited the phosphorylation of AKT and ERK and promoted the expression of Bax but inhibited Bcl 2. Compared with the control, both sorafenib and PNS downregulated the phosphorylation of AKT, ERK, and mTOR but sorafenib did not regulate the expression and phosphorylation of ATP1A1. Compared with the PNS groups, sorafenib had a more significant effect on AKT/ERK signaling pathways (Figure 4B). As shown in Figure 4C, PNS decreased the expression and phosphorylation of ATP1A1 in a dose-dependent manner, inhibiting the activation of AKT, ERK, and p38. PNS reduced the expression of PKC α but promoted the activation of PKC δ. The results of Q-PCR showed the same changes in the expression of AKT, ERK, and ATP1A1. The fluorescence intensity of ATP1A1 in HepG2 cells was decreased after PNS treatment and showed a dose-dependent manner (Figure 4D).

### Digitonin Decreased the Role of PNS on the Regulation of PI3K/AKT/mTOR and ERK Signaling Pathways, but Not the PKC Signaling Pathway

**FIGURE 5:**
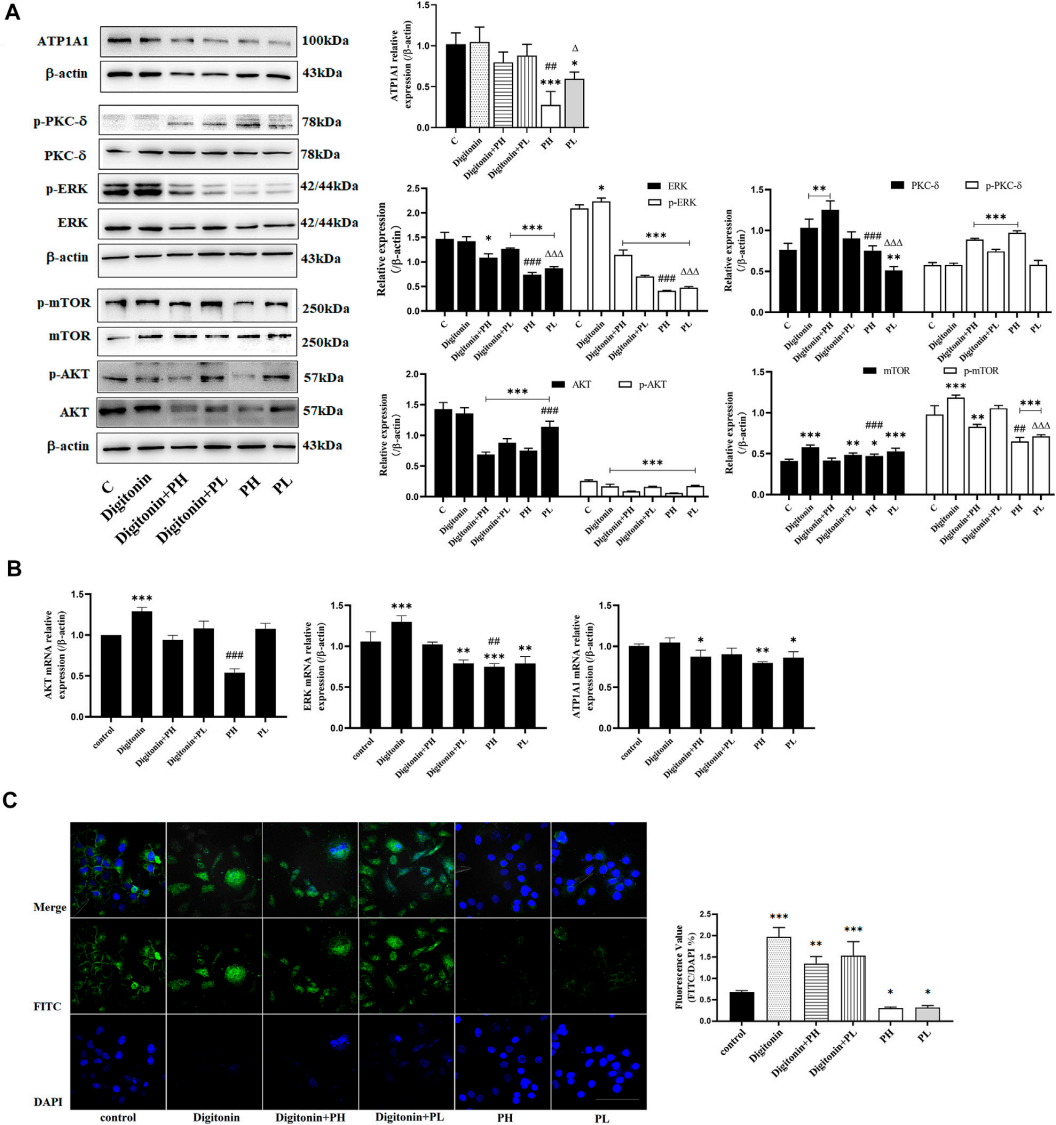
PNS and digitonin regulated the expression of proteins and mRNA of ATP1A1/AKT/ERK signaling pathways. Digitonin: 10 nmol/L; PH: 600 mg/L PNS; and PL: 300 mg/L PNS. **(A)** The representative image of Western blot after treatment with PNS and digitonin and their quantitative analysis are shown. **(B)** The relative expression of AKT, ERK1/2, and ATP1A1 by Q-PCR and their quantitative analysis are shown. **(C)** The fluorescence image of laser confocal detection of the expression of ATP1A1 on the HepG2 cell membrane (10 × 60). Scale bar = 50 μm. Data are expressed as mean ± standard deviation. *p < 0.05, **p<0.01, and ***p < 0.001 vs. the control group; ^Δ^ *p* < 0.05, ^ΔΔ^ *p* < 0.01, and ^ΔΔΔ^ *p* < 0.001 vs. the digitonin + PH group; ^#^ *p* < 0.05, ^##^ *p* < 0.01, and ^###^ *p* < 0.001 vs. the digitonin + PL group.

Low concentrations of digitonin block tumor cell growth without affecting the Na^+^/K^+^-ATPase activity (Fujii et al., 2018). The results showed that the expression of ATP1A1 in the PNS group was significantly reduced, but not in the digitonin group and in the cotreatment group (Figure 5A). The Western blot analysis showed that the expression and phosphorylation of AKT, mTOR, and ERK in HepG2 cells were inhibited after PNS treatment for 48 h. Digitonin attenuated the effect of PNS on AKT and ERK signaling pathways. The expression and phosphorylation of PKC δ were dramatically upregulated in all groups. qRT-PCR results showed similar results (Figure 5B). Fluorescence results showed that there was no complete nuclear morphology in the digitonin group and the relative fluorescence intensity of ATP1A1 did not decrease but increased (Figure 5C). The result of ATP1A1 expression in PNS co-treatment group was same in digitonin group. ATP1A1 was significantly reduced in PNS groups, and compared with the digitonin group, the amount of cell death was reduced.

### PNS Combined With Na^+^/K^+^-ATPase α1 Subunit

**FIGURE 6:**
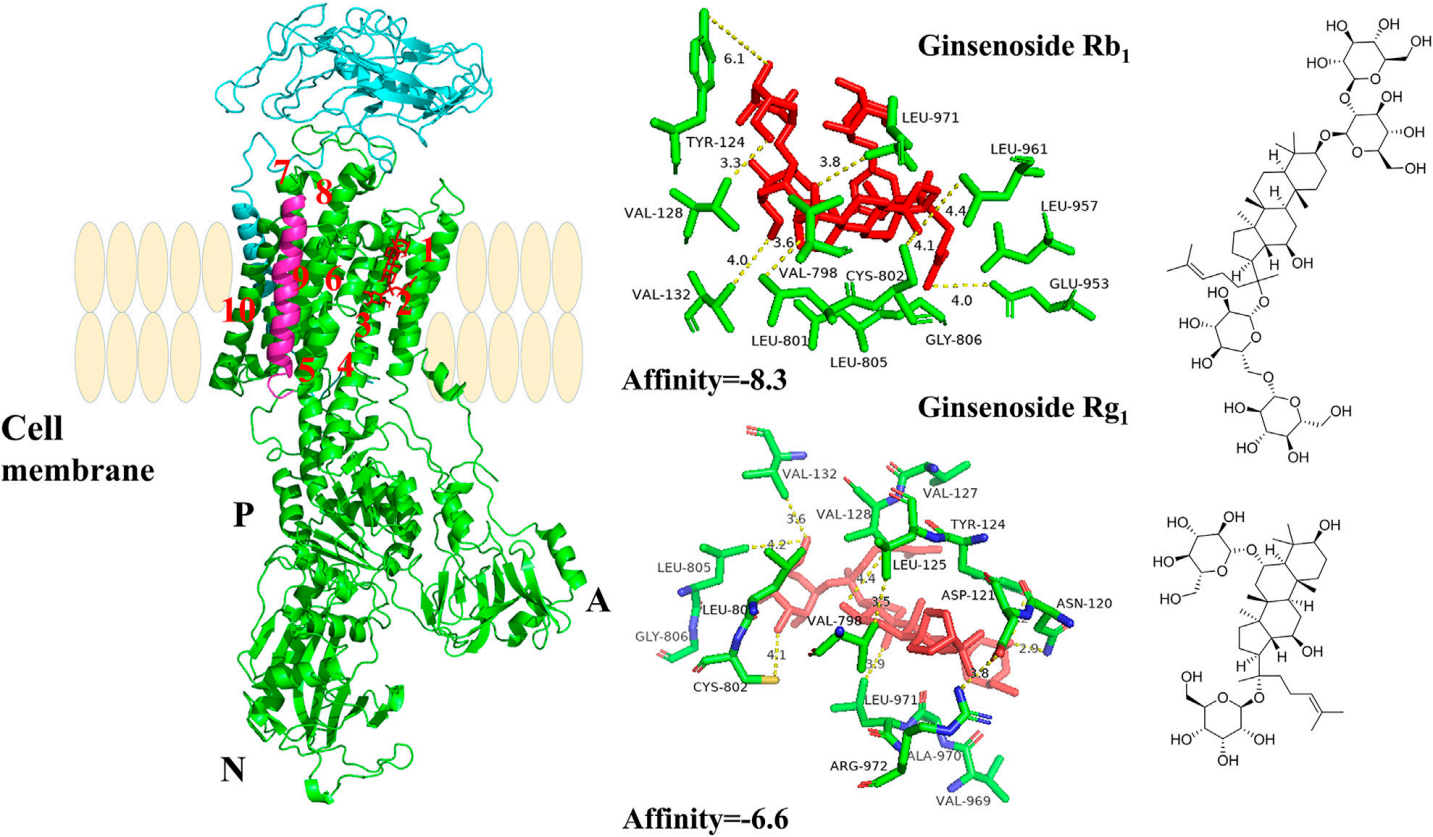
The crystal structure of the high-affinity Na^+^/K^+^-ATPase α1 subunit–ginsenoside Rb1 or –ginsenoside Rg1 complex. Ginsenoside Rb1 or ginsenoside Rg1 are depicted in red, and the chains of Na+/K+-ATPase α/β/γ subunits are represented by green, cyan blue, and magenta, respectively. Data deposition: the crystallography, atomic coordinates, and structure factors have been deposited in the Protein Data Bank (www.pdb.org (PDB ID code 3KDP)).

As shown in Figure 6, the α1 subunit was essentially involved in modulating the binding with cardiotonic steroids. The high affinity of Na^+^/K^+^-ATPase phosphoenzyme (E2P) was because of the transmembrane helices αM1–6 of the α subunit, forming an active pocket exposed to the outside of the cell. Rg_1_ and Rb_1_ mainly communicated with residues of αM1–4, such as VAL132, ASP121, and VAL798. The forces that they interacted with were predominantly the conjugation effect and H-bond force. The computational results suggested that the conjugated energy of Rb_1_ is less than that of Rg_1_ and more stable than that of Rg_1_.

## Discussion

*Panax notoginseng*, as a classic traditional Chinese medicine, has been widely used in clinical treatment, including trauma, cardiovascular disease, and cerebral ischemic stroke. As the main active components of *Panax notoginseng* (Burk.) F.H. Chen, PNS have multiple biological activities, including anti-inflammatory, antioxidative, and antitumor properties. Although the antitumor effect of PNS has been widely studied, the exact mechanism has not been fully elucidated. PNS could inhibit the tumor growth *in vivo* and inhibit proliferation and migration of tumor cells *in vitro*. The mechanisms are related to the regulation of PI3K/AKT/mTOR and NF-κB signaling pathways. However, how PNS regulate the signaling pathways has not yet been explained. Our present study focused on the effect of PNS on the ATP1A1 activity of HepG2 cells and the possible mechanism. ATP1A1 is a widely expressed membrane protein. Research studies had showed that ATP1A1 could be a novel therapeutic target for hepatocellular carcinoma. Based on the antitumor activity of PNS *in vivo* and *in vitro* and its regulatory effect on ATP1A1, we hypothesized that PNS could inhibit the proliferation and migration of HepG2 cells *via* the regulation of ATP1A1 transduction signaling and promote HepG2 cell apoptosis *in vitro*.

The expression of ATP1A1 in tumor tissues is higher than in surrounding tissues, and only about 30% of ATP1A1 participates in the function of NKAs. Most NKAs are involved in tyrosine kinase–dependent cell signal transduction, such as Src, of which ATP1A1 is the main participant in Src-mediated signal transduction (Liang et al., 2007; Zhuang et al., 2015). When knocking out a copy of the ATP1A1 gene, the expression of the α1 subunit will be reduced by 20–30%, resulting in at least a one-fold decrease in the activity of Src and MAPK (Chen et al., 2009; Lan et al., 2018). Src can regulate downstream proteins and cell functions (Wu et al., 2013), including PI3K/AKT, PLC/PKC, and JAK/STAT signaling pathways. In this study, we first observed that PNS could reduce the expression of ATP1A1 in tumor tissues of tumor-bearing mice. At the same time, we found that PNS downregulated the phosphorylation of AKT and ERK. *In vitro,* the results showed that the ATP1A1 expression was higher in HepG2 cells than in L02 cells. PNS could downregulate the expression of ATP1A1 and phosphate-ATP1A1 in L02 and HepG2 cells; meanwhile, PNS promoted the apoptosis of HepG2 cells and inhibited AKT/mTOR and ERK/MAPK activation. Sorafenib is a well-known drug used to treat HCC in clinics, as a Raf inhibitor, which significantly inhibits the phosphorylation of MEK and ERK, but not the expression of MEK and ERK, and inhibits the AKT signaling pathway. Compared with the control group, both sorafenib and PNS downregulated the phosphorylation of AKT, ERK, and mTOR. It was worth noting that sorafenib significantly reduced the expression of ERK and AKT, but not the expression and phosphorylation of ATP1A1. However, compared with the PNS groups, sorafenib had a more significant effect on the AKT/ERK signaling pathways. Combined with the antitumor effects of PNS, we suggested that one possible mechanism of PNS exerted the antitumor effect *via* inhibiting ATP1A1/AKT/MAPK signaling pathways *in vitro*.

The crosstalk between AKT and ERK signaling regulates each other and coregulates downstream signaling in certain cells (Lan et al., 2018). This study showed that PNS could inhibit the expression and phosphorylation of AKT and ERK and showed a dose-dependent effect. To better know the effect of PNS on the regulation of NKA, we used digitonin to inhibit the activation of NKA. We found that digitonin could significantly induce HepG2 cell lysis without affecting the expression of ATP1A1. When PNS were used in combination with digitonin, the expression of ATP1A1 was significantly downregulated compared with the digitonin group. The reasons were that digitonin reduced Na^+^/K^+^ exchange to cause cytomembrane depolarization by increasing intracellular Na^+^ concentration and reduced the dephosphorylation level of ATP to increase the permeability of the cytomembrane, promote nuclear fragmentation, and cause cell death (Robinson, 1980). The combined effect of the two drugs reduced the expression of ATP1A1 and promoted the death of HepG2 cells. Besides, digitonin attenuated the effects of PNS on the AKT/ERK signaling pathways. These observations implied that ATP1A1 played an important role in the regulation of AKT/ERK signaling pathways. Additional studies are also needed to determine the mutual regulation of ATP1A1 and the AKT/ERK signaling pathway and whether PNS can affect ATP1A1 in patients with HCC. The mechanism of PNS on tumors with a low expression of ATP1A1 needs to be further illustrated.

## Conclusion

Taken together, the study demonstrated for the first time that PNS could inhibit proliferation and migration of HepG2 cells by the regulation of ATP1A1 to affect AKT and ERK signaling pathways. The antitumor mechanism of PNS was related to suppressing the AKT and ERK signaling pathways to trigger apoptosis. Our findings will be helpful for further research and could provide a data basis for the clinical use of PNS.

## Data Availability

The original contributions presented in the study are included in the article/Supplementary Material; further inquiries can be directed to the corresponding authors.

## Abbreviations

AKT, protein kinase B; CTS, cardiac glycosides; ERK, extracellular signal–regulated kinase 1/2; EGFR, epidermal growth factor receptor; HCC, hepatocellular carcinoma; HepG2 cells, human hepatoblastoma HepG2 cells; H22, murine hepatoma-22; IC_50_, half-maximal inhibitory concentration; L02 cells, HL-7702 (L-02) normal human liver cells; MAPK, mitogen-activated protein kinase; mTOR, mechanistic target of rapamycin; NF-κB, nuclear transcription factor-κB; NKA, Na^+^/K^+^-ATPase sodium pump; PI3K, phosphatidylinositol 3-kinase; and Src, Src-family protein kinases.
