# Supplementary material for: Downregulation of ATP1A1 Expression by Panax notoginseng (Burk.) F.H. Chen Saponins: A Potential Mechanism of Antitumor Effects in HepG2 Cells and In Vivo
Source: Front Pharmacol. 2021 Oct 7;12:720368. doi: 10.3389/fphar.2021.720368 (PMC8529207; doi:10.3389/fphar.2021.720368)
Supplement: Supplementary file 1 [file DataSheet1.PDF]

Supplementary file

1. The quality control of PNS

We analyzed PNS by HPLC. Reference compounds were purchased from Desite Bio-tech Co. Ltd. (Chengdou, China). Notoginsenoside R_1 (Batch No: DST210415-050, Purity HPLC ≥ 98.0%), ginsenoside Rg_1 (Batch No: DSTDR000901, Purity HPLC ≥ 98.0%), ginsenoside Rb_1 (Batch No: DSTdr000601, Purity HPLC ≥ 98.0%), ginsenoside Rd (Batch No: DSTDR001501, Purity HPLC ≥ 98.0%). The standards and *P.notoginseng* saponin were dissolved in methanol, and the concentrations were 5 mg/mL and 20 mg/mL, respectively. We used Agilent SB-C18 column (250 × 4.6 mm, 5 μm, 30℃, UV detection is λ=203 nm, flow rate =1.5ml/min.), and determined the main components of PNS based on the HPLC chromatograms of standards and *P.notoginseng* saponis (Fig.S1).

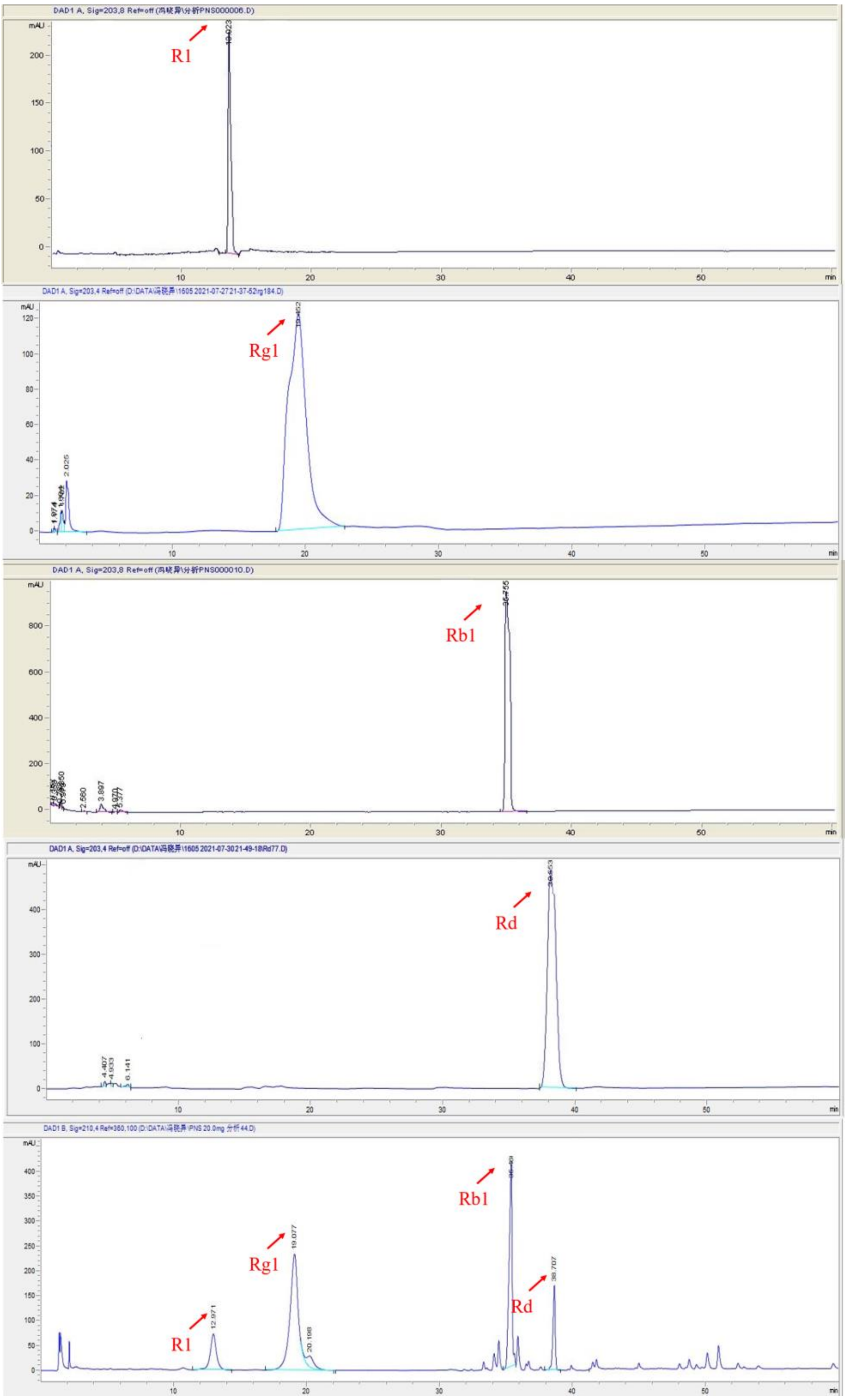

Fig.S1. Representative HPLC chromatogram of standards and *Panax notoginseng* saponin. Notoginsenoside R_1 (tr = 12.923 min), ginsenoside Rg_1 (tr = 19.452 min), ginsenoside Rb_1 (tr = 35.755 min), ginsenoside Rd (tr = 38.707 min). HPLC chromatogram was authenticated according to the Pharmacopoeia of the People's Republic of China identification key (2020, Volume 1). Column: Agilent SB-C18, 250 × 4.6 mm, 5 μm; Column temperature: 25℃; Flow rate =1.5ml/min; UV

detection is λ=203 nm. Gradient elution: acetonitrile (A) and Water (B): t 0min 20% A, t 20 min 20% A, t 45 min 46% A, t 55 min 55% A, 60 min 55% A. Injection volume: 10 μL.

2. *MTT assay*

HepG2 (1×10^4 cells/well) were incubated in 96-well plates in complete DMEM. Cells were treated with different concentrations of sorafenib (0, 0.63, 1.25, 2.5, 5, 10, 20 μmoL•L^{-1}) for 48 h. Cell viability was determined by MTT assay. Added 20 μL MTT to each well and incubated for 3 h. The formazan was dissolved in 150 μL of dimethyl sulfoxide (DMSO). Optical density was measured at 490 nm and cell viability was normalized as the percentage of control. Cell viability (%)=($OD_{control}$-OD_{sample}) / ($OD_{control}$-OD_{blank}) × 100%. IC_{50} (half maximal inhibitory concentration) is calculated by SPSS software. The IC_{50} was 13.09±0.83 μmoL•L^{-1}.

Table S1 Effects of sorafenib on cell viability of HepG2 cells ($\bar{x}\pm s$, n=3)

Group	Concentration （μmoL•L^{-1}）	Cell viability (%)
control	-	100.00±1.88
Sorafenib	0.625	100.99±4.21
	1.25	93.34±3.65
	2.5	77.82±1.68
	5	75.49±3.41
	10	55.56±5.62
	20	40.12±0.21
IC_{50}（μmoL•L^{-1}）		13.09±0.83
